# Childhood Exposure to Family Violence and Later-Life Sibling Relationships

**DOI:** 10.1093/geroni/igab046.284

**Published:** 2021-12-17

**Authors:** Jooyoung Kong, Jaime Goldberg

**Affiliations:** University of Wisconsin-Madison, Madison, Wisconsin, United States

## Abstract

There has been a growth in research examining the long-term effects of childhood adversity on later life outcomes; however, only a few studies have examined the impact that childhood adversity has on sibling relationships in late adulthood. To address this gap in the literature, the current study examines the latent class structure of childhood exposure to family violence and investigates whether a latent class membership is associated with aspects of later-life sibling relationships, including geographical proximity, frequency of contact, perceived closeness, similarity in outlook, and exchange of support. Using data from 3,921 adult participants in the Wisconsin Longitudinal Study and the Bolck, Croon, and Hagenaars (BCH) approach of latent class analysis (LCA), we identified five latent classes (prevalence rate noted): “Never experienced violence (75%),” “experienced sibling violence (7%),” “experienced parental abuse & witnessed domestic violence (4%),” “experienced father’s abuse & witnessed domestic violence (10%),” “experienced mother’s abuse & witnessed domestic violence (5%)”. Childhood exposure to family violence was a significant predictor of later-life sibling relationships. Specifically, adults who experienced sibling violence and their mother’s and/or father’s abuse in childhood showed significantly lower perceived closeness and similarity in outlook with their siblings in adulthood than those who did not experience violence. Our findings suggest that childhood exposure to family violence may have a long-term negative impact on the emotional aspect of sibling relationships. Future research may explore how the impact of childhood adversity on sibling relationships affects other aspects of adult lives, such as individual well-being or caregiving for aging parents.

